# Energy and economic efficiency of climate-smart agriculture practices in a rice–wheat cropping system of India

**DOI:** 10.1038/s41598-022-12686-4

**Published:** 2022-05-24

**Authors:** S. K. Kakraliya, H. S. Jat, Ishwar Singh, M. K. Gora, Manish Kakraliya, Deepak Bijarniya, P. C. Sharma, M. L. Jat

**Affiliations:** 1 CCS Haryana Agricultural University, Hisar, Haryana 125004 India; 2 International Maize and Wheat Improvement Center (CIMMYT), NASC Complex, Pusa, New Delhi, 110012 India; 3 ICAR-Central Soil Salinity Research Institute, Karnal, Haryana 132001 India

**Keywords:** Climate sciences, Ecology, Environmental sciences

## Abstract

Intensive tillage operations, indiscriminate use of irrigation water, chemical fertilizers, and pesticides and crop biomass burning have made the conventional rice–wheat (RW) system highly energy-intensive and inefficient. In the recent past, portfolios of climate-smart agricultural practices (CSAP) have been promoted as a potential alternative to improve the energy efficiency in conventional RW system. Therefore, to evaluate the energy input–output relation, energy flow and economic efficiency in various combinations of crop management options, a 3-year (2014–2017) on-farm study was conducted at Karnal, India. Various portfolio of management practices; Sc1-Business as usual (BAU) or Conventional tillage (CT) without residue, Sc2-CT with residue, Sc3-Reduce tillage (RT) with residue + recommended dose of fertilizer (RDF), Sc4-RT/Zero tillage (ZT) with residue + RDF, Sc5-ZT with residue + RDF + GreenSeeker + Tensiometer, Sc6-Sc5 + Nutrient expert were investigated. Present study results revealed that net energy, energy use efficiency and energy productivity were 11–18, 31–51 and 29–53% higher under CSAP (mean of Sc4, Sc5 and Sc6) in RW system than Sc1, respectively. However, renewable and non-renewable energy inputs were 14 and 33% higher in Sc1 compared to CSAP (4028 and 49,547 MJ ha^−1^), respectively, it showed that BAU practices mostly dependents on non-renewable energy sources whereas CSAP dependents on renewable energy sources. Similarly, the adoption of CSAP improved the biomass yield, net farm income and economic efficiency by 6–9, 18–23 and 42–58%, respectively compared to Sc1. Overall, the adoption of CSAP could be a viable alternative for improving energy use efficiency, farm profitability and eco-efficiency in the RW system.

## Introduction

Rice (*Oryza sativa* L.)–wheat (*Triticum aestivum*L.) rotation in Indo-Gangetic Plains (IGP) of South Asia, with ~ 13.5 million hectares (Mha) acreage, is the backbone for food supply^1–3^. In India, the belt of rice–wheat (RW) rotation occupies almost 10.5 Mha areas and is the main source of food, nutrition and livelihood security in the country^1,3^. In RW system of the IGP, the energy is expensed in several forms such as labour, animal draft, farm machines, inorganic fertilizers, insecticides, fungicides and herbicides, electricity for pumping irrigation water, manual transplanting of rice seedlings into the well-puddled soils (puddled transplanted rice; PTR) etc. But presently, RW system are showing energy insecurity in the IGP`s region due to intensive energy used in various crop production activities such as multiple tillage (2–3 dry harrowing, 1–2 pass of rotavator/cultivators, 2–3 wet harrowing in rice and 1–2 planking) to get ready the field for rice and wheat planting^2,4^. Further, the use of more manual labour in transplanting of rice seedlings (30 days old age) into well-puddled soil also consumes an enormous amount of energy. In PTR, puddling alone needs approximately 25–30% of the total irrigation water requirement of rice^2^. Higher water requirement in rice is also due to more water losses in the form of puddling, percolation and surface evaporation which ultimately leads to more consumption of electricity for groundwater pumping for puddling (wet harrowing), nursery raising and frequent irrigation to keep the fields flooded throughout the growing season^2,4^. In upper and middle IGP, irrigation water is mostly driven by electricity pumps whereas in lower IGP diesel pumps are mainly used, and both consume a huge quantum of energy^2^. In recent years, higher fertilizer use, pumping of groundwater and depletion of groundwater resources along with higher pesticide consumption made RW system energy intensive which is the major threat to its future sustainability^5^. Approximately 84% of wheat production costs incurred from these energy-intensive inputs (*e.g.,* irrigation, land preparation and fertilizers)^6,7^. In South Asia and elsewhere, published outcomes from diverse research findings have highlighted that intensive tillage practices accounts ~ 25% or more of the total production cost in RW system^1^. This energy-intensive system has started suffering from other production fatigue owing to over mining of nutrients, declining factor productivity, increasing production cost, reducing farm profitability, deteriorating soil health and labour shortage causing concern about its sustainability^2,8^. Escalating the production and energy costs in the RW system are not only harmful to keeping productivity and farmers' farm incomes but are also a major challenge for global food and energy security^8–10^.

Recently, under the situation of intensification of existing cropping systems the energy–farming relationship is becoming more vital^11^. With the adoption of traditional practices and indiscriminate use of available resources/production inputs use of the energy resources has greater than before remarkably; therefore, to reduce the energy consumption in agriculture while sustaining the food production in this densely populated region, there is a need to be switched over towards more energy-efficient crop management practices. In recent past, various energy-smart agricultural practices have been identified and validated for energy-intensive traditional practices in RW rotation^1,11^.

Energy smart agriculture (ESA) practices namely laser land levelling, zero tillage (ZT), direct-seeded rice (DSR), site-specific nutrient management (SSNM) and precision irrigation management have been suggested as potentially sustainable alternatives to traditional energy-intensive practices. Non-requirement of intensive tillage operations in energy-smart agriculture translates into less diesel requirement, lesser working time and slower depreciation rates of equipments. These all are reducing energy inputs in various farm operations, particularly from land preparation, as well as from the agricultural machinery manufacturing processes^6^. By adopting the ESA-based ZT system under the RW system, farmers could save 36 L diesel ha^-1^ which is equivalent to 2027 MJ ha ^−1^. In addition, energy-intensive agricultural practices have high carbon footprints especially, greenhouse gases (CO_2_, N_2_O, CH_4_, etc.)^12^, have enhanced the global energy budget by more than 10 times since the beginning of twentieth century^13^and at the same time increased the cost of cultivation in crop production by approximately 4 times than ZT farming during the same period^14^. Therefore, energy requirements can be minimized by adopting of energy-efficient technologies. Furthermore, adequate availability of the accurate source of energy and its effective and proficient use are the prerequisites for the conventional RW system with the lowest energy inputs^12^. In energy budgeting, it is essential to identify or develop energy-efficient technologies, with less energy and environmental footprints. A number of climate smart agriculture (CSA) practices have been assessed in cereal systems as an alternative to energy-intensive traditional practices. So far, information on energy footprints of these practices together (as a portfolio) is scanty. Hence, there is an urgent need for a scientific assessment to use a holistic tactic of principles and procedures known to increase the energy-use efficiency (EUE) and decrease the input energy as well as associated carbon footprints in crop production.

In the IGP of South Asia, most research reported only productivity, profitability and water use efficiency under RW rotation. Thus, the present on-farm multi-location participatory research was carried out within climate-smart villages of Haryana (India) for 3-years to test the hypothesis that CSA improves the EUE, decreases the carbon footprints, cost of production and efficient use of production inputs in the RW system without jeopardizing the productivity of the crops relative to those for the conventional management practice of the RW production system, and offers a hygienic and environmentally sustainable energy use efficient production technology for this IGP region of India. The key objectives of the study were to: (1) to find out the energy conservation and energy-efficient agricultural practices for the RW system in western IGP of India; (2) to assess the key energy indicators and inputs for the RW system; and (3) to study the economic feasibility of most energy-efficient practices in the RW system.

## Results and discussion

### Source and operation-wise energy utilization pattern

#### Field operations/seedbed preparation

**Figure 1:**
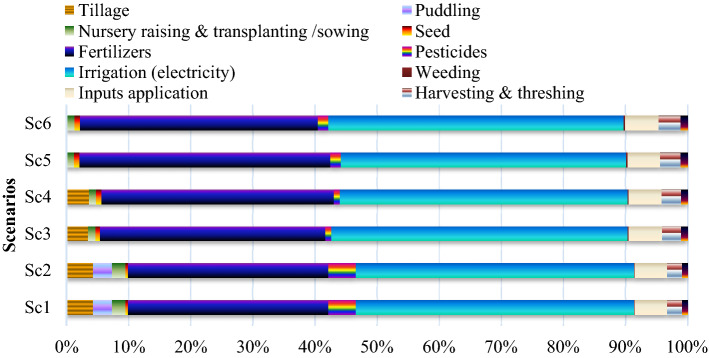
Operation-wise input energy-use pattern (%)under different management practices in rice. Where; Sc1, business as usual-conventional tillage (CT) without residue; Sc2, CT with residue; Sc3, reduce tillage (RT) with residue + recommended dose of fertilizer (RDF); Sc4, RT/Zero tillage (ZT) with residue + RDF; Sc5, ZT with residue + RDF + GreenSeeker + Tensiometer; Sc6, Sc5 + Nutrient expert.

Energy used in different field operations under various crop management activities was significantly affected by the rice establishment methods and was ranged from 422 to 436 MJ ha^−1^ (Table 1 and Fig. 1, S2). Business as usual (Sc1) with high energy intensive practices consumed the highest (4336 MJ ha^−1^) energy in seed bed preparation, whereas in Sc5 and Sc6 no energy was required for seed bed preparation (Fig. 1). CSAP (mean of Sc4, Sc5 and Sc6) consumed 57% less energy in crop establishment (transplanting/sowing) operations compared Sc1 (978 MJ ha^−1^). Irrespective of field operations, tillage consumed highest input energy in conventional management practice of RW system. This was due to repeated (5–6 passes) dry and wet tillage to prepare a seedbed for nursery raising and puddling consumed more diesel in machinery in Sc1. In addition to this, Sc1 and Sc2 required 15–20 additional manual labour for transplanting rice seedlings.

**Table 1:** Energy (MJ ha^−1^) utilization pattern under different management practices in rice and wheat (mean of 3-years).

Scenarios	Field operations			Agronomic inputs				Labor		Harvesting & threshing	Transportation
	Tillage	Puddling	Sowing/transplanting	Seed	Fertilizers	Pesticides	Irrigation	Weeding	Input application		
**Rice**											
Sc1	1977	1381	978	184	14,748^A^	1993	20,471^A^	47.04	2394^A^	1126	385
Sc2	1977	1381	978	184	14,748^A^	1993	20,471^A^	47.04	2394^A^	1126	385
Sc3	1310	0	422	294	13,474^B^	351	17,727^B^	62.72	2011^B^	1126	385
Sc4	1310	0	422	294	13,474^B^	341	16,692^C^	62.72	1896^C^	1126	385
Sc5	0	0	422	294	13,637^B^	541	15518^D^	62.72	1765^D^	1126	385
Sc6	0	0	422	294	12,491^C^	541	15,518^D^	62.72	1765^D^	1126	385
**Wheat**											
Sc1	2228	-NA-	850	1470	14,328^A^	364	3928^A^	16	471^A^	845	369
Sc2	2228	-NA-	850	1470	14,328^A^	364	3928^A^	16	471^A^	845	369
Sc3	1382	-NA-	594	1470	12,752^B^	352	3831^A^	16	453^A^	845	369
Sc4	0	-NA-	892	1470	12,752^B^	462	3236^B^	0	393^B^	845	369
Sc5	0	-NA-	892	1470	11,597^C^	462	3236^B^	0	393^B^	845	369
Sc6	0	-NA-	892	1470	10,809^D^	462	3236^B^	0	393^B^	845	369

Values with different Upper case (A–D) letters are significantly different between each scenarios at *p* < 0.05 (Duncan multiple range tests for separation of mean).

Sc1, business as usual or conventional tillage (CT) without residue; Sc2, CT with residue; Sc3, reduce tillage (RT) with residue + recommended dose of fertilizer (RDF); Sc4, RT/Zero tillage (ZT) with residue + RDF; Sc5, ZT with residue + RDF + GreenSeeker + Tensiometer; Sc6, Sc5 + Nutrient expert.

**Figure 2:**
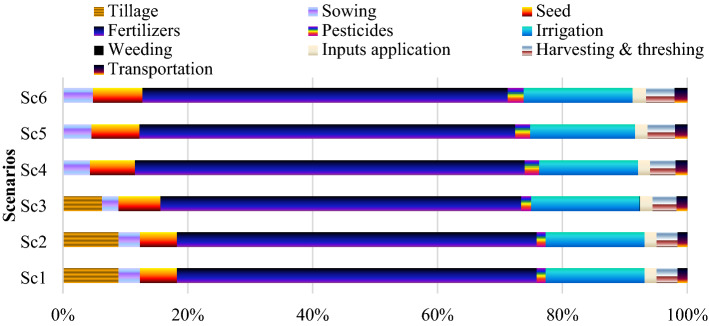
Operation-wise input energy-use pattern (%) under different management practices in wheat. Where; Sc1, business as usual or conventional tillage (CT) without residue; Sc2, CT with residue; Sc3, reduce tillage (RT) with residue + recommended dose of fertilizer (RDF); Sc4, RT/Zero tillage (ZT) with residue + RDF; Sc5, ZT with residue + RDF + GreenSeeker + Tensiometer; Sc6, Sc5 + Nutrient expert.

In wheat, energy used under different management practices for seedbed preparations ranged from 892 to 3078 MJ ha^−1^ and were significantly affected by crop establishment method (Table 1). In seedbed preparation, Sc1 and Sc2 consumed highest energy (2228 MJ ha^−1^) followed by Sc3 (1382 MJ ha^−1^), whereas in Sc5 and Sc6 no energy was required for seed bed preparation. Sc3-Sc6 consumed ~ 53% less energy in seedbed preparation and in sowing compared to Sc1 (Fig. 2). Business as usual (Sc1) consumed more energy because of it required more tillage operations in seedbed preparation^1,4^. However, in CSAP, tillage is not required for seeded preparation and energy is used only for seed sowing.

**Figure 3:**
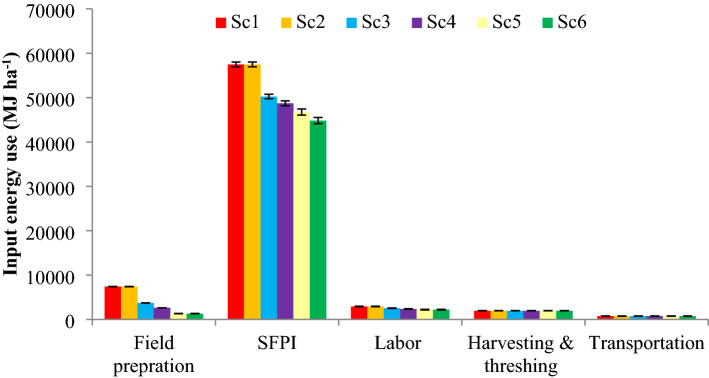
Operation-wise input energy-use (%) of RW system under different management practices. Where; SFPI are seed, fertilizer, pesticides and irrigation. Sc1, business as usual-conventional tillage (CT) without residue; Sc2, CT with residue; Sc3, REDUCE tillage (RT) with residue + recommended dose of fertilizer (RDF); Sc4, RT/Zero tillage (ZT) with residue + RDF; Sc5, ZT with residue + RDF + GreenSeeker + Tensiometer; Sc6, Sc5 + Nutrient expert. Vertical bars indicate ± S.E. of mean of the observed values.

On the system basis, CSAP consumed 76% less energy in seed bed preparation compared to Sc1 (7416 MJ ha^−1^) (Fig. 3). The higher energy consumption in tillage could be due to fewer usages of modern agricultural machineries and higher use of human & animal power in conventional RW production (Fig. 3). These findings are in support of many other researchers they revealed that diesel consumption (15–20 L ha^−1^) can be reduced by minimizing numbers of tillage operations^5,6^. Gathala et al*.*^9^ and Laik et al*.*^11^ have also described that more tillage operations are the biggest energy consumer (~ 40% of the total energy) compared to best agronomic management practices.

#### Seed, fertilizers, pesticides and irrigation (SFPI)

In rice production, agronomic energy inputs (SFPI) consumed ~ 84% of the total energy inputs, of which irrigation alone consumed about 46% (mean of six scenarios’ total energy input 3,8483 MJ ha^−1^) (Table 1 and Fig. 1, S2). Sc1 (puddled transplanted rice; PTR) consumed 29% higher energy in irrigation compared to CSAP (direct seeded rice; DSR) (Fig. 1). This was due to more electricity consumption in lifting of irrigation water from borewell for nursery raising, puddling operations and continuous flooding of water to complete the life cycle of crops. Furthermore, inorganic fertilizers were the second most important input that accounted for ~ 36% of total energy. Chaudhary et al*.*^4^and Pathak et al*.*^15^ stated that out of the total energy, about 43% energy is required for irrigation and fertilizers in rice production. The CSAP consumed 76, 22 and 11% less energy in pesticides, irrigation and fertilizer, respectively compared to Sc1 (Fig. 1). However, the seed energy was lower in Sc1 (transplanting methods) of rice production than CSAP (DSR), since the seed rate was used lower in PTR; these results were in accordance with Chaudhary et al.^4^ and Yuan et al*.*^12^. Similarly, CSAP (DSR) recorded 87% more energy for weed control and inter-cultivations than Sc1 (PTR), due to use of higher amount of herbicides in DSR (Sc3–Sc6). While in PTR (Sc1 and Sc2), submergence of water minimized the weed problem, which contributed to lesser use of herbicides. Nevertheless, the energy savings in various interculture operations and weed management practices under PTR weren't enough to compensate its more energy consumption in nursery raising, puddling for rice seedling transplantation and irrigation. Overall, Sc6, Sc5, Sc4 and Sc3 consumed 23, 20, 18 and 15% less energy in SFPI compared to Sc1 (37,212 MJ ha^−1^) (Fig. 1). Laik et al*.*^11^ and Nassiri et al*.*^16^ results are validated by those who reported the highest energy consumption in conventional RW production system compared to CA based RW system.

Like rice, in wheat production also, agronomic energy inputs/SFPI were the major energy consumers that contributed nearly 84% energy out of the total energy (21,660 MJ ha^−1^) (Table 1). Among the agronomic inputs (SFPI), fertilizer (F) was the foremost energy input requiring about 70% energy (18,208 MJha^−1^) of the total energy. Furthermore, irrigation is the second major energy consumer that contributed around 16% of the total agronomic energy inputs (Table 1 and Fig. 2). Overall, CSAP consumed 18.2 and 17.6% lesser energy in fertilizer and irrigation respectively, compared to Sc1 (14,328 and 3928 MJ ha^−1^) (Fig. 2). Less fertilizer and irrigation requirement under CSAP was due to precision agronomic input management, whereas, in Sc1 more use of N fertilizer and irrigation was made it more energy intensive. However, CSAPs consumed 26% higher energy in pesticides than to Sc1 (364 MJ ha^−1^). Sc6, Sc5, Sc4 and Sc3 consumed 20, 17, 11 and 8% less energy under SFPI compared to Sc1 (20,090 MJ ha^−1^). The findings of the present study are in accordance with some researchers^12^. On the system basis, CSAP consumed 19% lower energy under agronomic inputs/SFPI compared to Sc1 (57,485 MJ ha^−1^) (Fig. 3).

#### Crop managements, harvesting and threshing

The energy utilization pattern for rice production in different crop management operations (intercultural, weeding and inputs application) are presented in Table 1 and Fig. S2. In 3-years, CSAP consumed 23% less energy under various crop management activities compared to Sc1 (2394 MJ ha^−1^). Among the crop management practices, CSAP consumed 33% higher energy in weeding operation compared to Sc1 in rice production (Fig. 1). Likewise, in wheat production, Sc6 and Sc5 computed 19% less energy in crop management activities compared to Sc1 (487 MJ ha^−1^). Sc1, Sc2 and Sc3 consumed 15.6 MJ ha^−1^ higher energy in weeding operations whereas, no energy required in weeding under CSAP (mean of Sc4, Sc5 and Sc6) (Table 1). The similar energy use pattern was recorded under all scenarios for harvesting and threshing operations in both the crops (Fig. 2). In RW system, CSAP and Sc3 consumed 23 and 13% less energy in input application compared to Sc1 (2264 MJ ha^−1^), respectively (Fig. 3). The highest energy use in various crop management practices under Sc1 was due to more energy required for the application of fertilizers, pesticides, hand weeding and inter-culture operations compared to CSAP. Findings of current study are in accordance who also recorded that smart crop management practices required less energy compared to conventional practices^4,5,12,17^.

#### Direct–indirect and renewable–non-renewable energy

In rice production, direct and non-renewable energy consumption was more than indirect and renewable energy (Table 2). Direct energy in different cultivation methods of rice was in the range of 57–63%, whereas indirect energy was 37–43% of total energy consumed. Among the direct energy sources, application of irrigation water in all scenarios of rice cultivation consumed the highest direct energy, which showed that irrigation methods in rice cultivation should be standardized with low water use for its future sustainability. The findings of past researchers highlighted that more tillage operation before planting needed around 1/3^rd^ of the total field operational energy, and that can be saved without affecting the crop yields with the adoption of zero tillage based rice cultivation practices^6,9,15,18^. CSAP (mean of Sc4, Sc5 and Sc6) recorded 43 and 17% less consumption of direct energy & indirect energy in rice cultivation compared to Sc1 (19,264 and 5735 MJ ha^−1^), respectively. The Sc3 also recorded 20 and 17% less consumption of direct & indirect energy compared to Sc1, respectively (Table 2). The contrast effects (BAU vs CSAP and I-BAU vs CSAP) were significant for direct and indirect energy (Table S2). However, BAU versus I-BAU was not-significant for direct energy but significant for indirect energy.

**Table 2:** Total energy input (MJ ha^−1^) in the form of direct, indirect, renewable and non-renewable energy for different management practices under the rice, wheat and RW system.

Scenarios	Direct energy			Indirect energy			Renewable energy			Non-renewable energy		
	Rice	Wheat	RW system	Rice	Wheat	RW system	Rice	Wheat	System	Rice	Wheat	RW system
Sc1	28744^A^	8707^A^	37,452^A^	16,925^A^	16,162^A^	33087^A^	2624^A^	1957^A^	4582^A^	43,045^A^	22,912^A^	65,957^A^
Sc2	28744^A^	8708^A^	37,452^A^	16,925^A^	16,162^A^	33,087^A^	2624^A^	1957^A^	4582^A^	43,045^A^	22,912^A^	65,957^A^
Sc3	23029^B^	7490^B^	30,519^B^	14,120^C^	14,574^B^	28,694^B^	2368^B^	1939^A^	4306^B^	34,781^B^	20,125^B^	54,906^B^
Sc4	21879^C^	5985^C^	27,864^C^	14,110^C^	14,684^B^	28,794^B^	2253^C^	1863^B^	4115^C^	33,736^C^	18,806^C^	52,542^C^
Sc5	19264^D^	5735^C^	24,999^D^	14,473^B^	13,529^C^	28,002^C^	2122^D^	1863^B^	3985^D^	31,615^D^	17,401^D^	49,016^D^
Sc6	19264^D^	5735^C^	24,999^D^	13,326^D^	12,741^D^	26,068^D^	2122^D^	1863^B^	3985^D^	30,468^E^	16,613^E^	47,082^E^

Values with different upper case (A–E) letters are significantly different between each scenarios at *p* < 0.05 (Duncan multiple range tests for separation of mean).

Sc1, business as usual-conventional tillage (CT) without residue; Sc2, CT with residue; Sc3, reduce tillage (RT) with residue + recommended dose of fertilizer (RDF); Sc4, RT/Zero tillage (ZT) with residue + RDF; Sc5, ZT with residue + RDF + GreenSeeker + Tensiometer; Sc6, Sc5 + Nutrient expert.

The contribution of renewable energy was very low in rice cultivation methods and it highlighted that the cultivation of rice is mainly based on non-renewable sources^4,5,11,15^. In our study, higher percent of electricitical energy consumed for water pumping from tube-wells, could be owing to less charges of electricity in Haryana, India^19–21^. In the study`s area, electric energy consumed in crop production is generated mostly from non-renewable sources, particularly fossil fuels. Furthermore, non-renewable sources are still the main fuel in power plants. The contrast effect (BAU vs CSAP) was significant for renewable and non-renewable energy (Table S2).

In wheat cultivation methods, indirect & non-renewable energy consumption was greater than the direct & renewable energy. Less renewable energy uses in wheat cultivation showed that wheat production is mainly based on non-renewable resources. CSAP recorded 52 and 19% less direct and indirect energy in wheat cultivation compared to Sc1, respectively (Table 2).

In RW system, direct and indirect energy consumption varied from 24,999 to 37,452 MJ ha^−1^ and 26,068 to 33,087 MJ ha^−1^, respectively (Table 2). Business as usual required more direct energy (diesel in field operations, electricity in irrigation and labour in crop management) than indirect energy in the CT-based RW system. However, CSAP required less direct energy compared to indirect energy, which showed that less number of field operations are required under CSA-based RW production system. The contrast effect (BAU vs CSAP) were significant to direct and indirect energy (Table S2).

In RW system, higher renewable & non-renewable input energy was recorded under Sc1 and Sc2 (4582 and 65,957 MJ ha^−1^) followed by Sc3 (4306 and 54,906 MJ ha^−1^) as compared to CSAP (3985 and 47,082 MJ ha^−1^) (Table 2). The contrast effects were significant to renewable & non-renewable energy (Table S2). Present study indicated that conventional RW production system in the IGP plains are mostly dependent on non-renewable energysources^4,15,20,22^. Overall, non-renewable energy through fuel, electricity for ground water, inorganic fertilizers, pesticides and farm machineries shared maximum energy inputs followed by renewable resources viz*.,*labour, tractor, seed, etc.^11,15,18^. Dependence on non-renewable energy impacted the sustainability of the RW system^15^. Noteworthy, renewable energy is eco-friendly as well as reliable source of energy; hence, the use of renewable energy highlighted huge benefits, counting lesser contributions to greenhouse gasses emissions and enhanced environmental quality^5^. The present findings highlighted that more focus should be kept to improve, renewable energy use, technical innovation and optimized investment in rice and wheat production.

#### Energy balance sheet (input–output and net energy)

The total energy used for various rice production methods varied from 32,606 to 45,685 MJ ha^−1^ and was significantly affected by different crop management practices (Table 3). Our study results are in track with those of other similar research studies conducted in the IGP region for RW system^4,5,11^. Among the different rice production methods, PTR cultivation method (Sc1) of rice noted higher energy input than the CSAP (DSR method). Sc1 (32,606 MJ ha^−1^) recorded 40, 35, 27 and 23% higher energy use in rice production over Sc6, Sc5, Sc4 and Sc3, respectively (Table 3). Similarly, Sc1 (32,606 MJ ha^−1^) recorded 35, 29, 22 and 13% higher energy use in wheat production over Sc6, Sc5, Sc4 and Sc3, respectively. The CSAP and Sc3 used 24 and 16% less energy under RW system compared to Sc1 (70,538 MJ ha^−1^), respectively. However, CSAP recorded higher energy output from rice, wheat and RW system compared to Sc1. Compared to Sc1, the CSAP produced 1, 14 and 6% higher grain output energy under rice, wheat and system, respectively. The minimum input and maximum output energy under Sc6 were due to gained more net energy for both the crops during the respective years (Table 3). Linear contrast effects were significant to total energy input in rice, wheat and RW production systems. However, contrast effects were not significant to energy input in rice and RW system but significant to wheat production system.

**Table 3:** Energy (MJ ha^−1^) balance under different management practices in rice, wheat and RW system (mean of 3 years).

Scenarios	Total energy input			Energy output			Net energy			Energy use efficiencyc			Grain energy productivity		
	Rice	Wheat	RW system	Rice	Wheat	RW system	Rice	Wheat	RW system	Rice	Wheat	RW system	Rice	Wheat	RW system
Sc1	45,685	24,869	70,538^A^	217,461^B^	153,780^C^	371,241^C^	171,792^E^	79,378^C^	300,703^D^	4.77^D^	6.19^E^	5.27^D^	0.15	0.21	0.17
Sc2	45,685	24,869	70,538^A^	220,720^B^	156,036^BC^	376,756^C^	175,051^DE^	80336^BC^	306,218^D^	4.84^D^	6.28^E^	5.35^D^	0.15	0.21	0.17
Sc3	37,165	22,063	59,212^B^	217,292^B^	162,005^B^	379298^C^	180,144^CD^	82,831^B^	320,085^C^	5.86^C^	7.34^D^	6.41^C^	0.18	0.24	0.21
Sc4	36,005	20,669	56,657^C^	221,854^AB^	169,805^A^	391,659^B^	185,865^BC^	86,971^A^	335,002^B^	6.19^BC^	8.22^C^	6.92^B^	0.19	0.28	0.22
Sc5	33,753	19,264	53,001^D^	222,944^AB^	173,759^A^	396,704^AB^	189,208^AB^	88,872^A^	343,703^AB^	6.64^AB^	9.03^B^	7.51^A^	0.20	0.30	0.24
Sc6	32,606	18,476	51,067^E^	228,562^A^	175,866^A^	404,428^A^	195,972^A^	89,906^A^	353,362^A^	7.05^A^	6.19^E^	7.94^A^	0.22	0.32	0.26

Values with different upper case (A–E) letters are significantly different between each scenarios at *p* < 0.05 (Duncan multiple range tests for separation of mean).

Sc1, business as usual-conventional tillage (CT) without residue; Sc2, CT with residue; Sc3, reduce tillage (RT) with residue + recommended dose of fertilizer (RDF); Sc4, RT/Zero tillage (ZT) with residue + RDF; Sc5, ZT with residue + RDF + GreenSeeker + Tensiometer; Sc6, Sc5 + Nutrient expert.

In rice production, the energy saving under CSAP was due to less energy inputs used in electricity that was associated with less irrigation water use in cultivation^4,5,11^. Efficient water management practice had a positive effect on energy consumption^5,11^and diverse energy sources across water regimens in India^1,12,16^. Our study showed that the energy input in existing rice and wheat production can be further minimized with precision water management techniques and, optimization of irrigation water management based on the precision land-levelling, frequent irrigation in rice, tensiometer based irrigation and zero tillage can efficiently decrease the total energy consumption in the IGP of India^2,11^.

On an average, fertilizer was the first and second largest source of energy consumption in rice and wheat in all scenarios (Figs. 1 and 2), respectively. Aggregate proof from the current study and other similar studies highlighted that fertilizer consumption created the major share of the total energy input in crop production^10–12^. Among different fertilizers, N-fertilizers consumed the most energy input and constituted 94% in Sc1 and 87% in CSAP of the energy from fertilizers in RW system. From several past evidences, it is crystal clear that fertilizer application is exceeded to the highest demand for crop growth & development in this region, that further encouraged low resource use efficiency (RUE) and higher environmental footprints^23,24^. Thus, it is necessary to use fertilizers efficiently to reduce energy use and to prevent environmental degradation. Overall, the higher energy input was allied with more tillage, labour, irrigation and higher use of N-fertilizers in Sc1 compared to CSAP. Erenstein et al*.*^6^, Gathala et al*.*^9^and Ladha et al*.*^3^ also described that more tillage for seed bed preparation, more number of irrigation, higher labour and higher fertilizer inputs are the main interventions for higher energy usage under traditional farming. The higher output energy of rice, wheat and RW system with CSAP might be due to the multiple effects of applied nutrients^1^, zero tillage^5^, residue management, improved soil health^2^, good water regimes^5,11^and improved nutrient use efficiency (NUE) relative to Sc1. The CSAP recorded greater crop yields that ultimately reflected to greater net energy, EUE, human energy profitability, EP, over conventional methods of RW system.

#### Energy use efficiency (EUE) and productivity

Energy use efficiency is an index used to measure the amount of energy that is effectively used in different farm activities. The highest input and the lowest output energy under Sc1 resulted into the lowest EUE and energy productivity (EP). Contrarily, the lowest energy input and the highest energy output under CSAP (mean of Sc4, Sc5 and Sc6) resulted into the maximum EUE and EP in both the crops in all the study’s years (Table 3). The average energy use efficiency was 52, 53 and 54% higher under Sc6 in rice, wheat and RW system compared to Sc1 (Table 3), respectively. CSAP recorded 44% (7.57 MJ MJ^−1^) higher EUE compared to Sc1 (5.28 MJ MJ^−1^) in the RW system. Linear contrast effects were also significant to EUE in rice, wheat and RW production systems. The large gap among the two values was due to tillage, irrigation and fertilizers which highlighted that EUE can be enhanced with reduced tillage, precision use of irrigation water and nutrient. Remarkably, the values observed in the current finding fall around the range described by other researchers^11^ who revealed that the EUE of RW production in IGP ranged 3.94 ± 1.31 MJ MJ^−1^. Overall, the results of the current study showed that those existing production methods of the RW system in IGP are not too efficient. Besides, RW system is damaging to agro-ecosystems because of imbalance and excess use of inputs. Hence, efficient use of production inputs would be helpful in optimizing energy consumption in RW system in the IGP region of South Asia.

Energy productivity (EP) was statistically higher in the Sc6 in rice (0.15 kg MJ^−1^), wheat (0.21 kg MJ^−1^) and RW system (0.17 kg MJ^−1^) than in the Sc1 (Table 3). These findings revealed that an additional ~ 27% of RW system yield was gained per unit energy input in the Sc6 compared with the other scenarios (0.20 kg MJ^−1^). CSAP recorded 40% higher EP compared to Sc1 (0.17 kg MJ^−1^) in RW system. Linear contrast effects were significant to EP in rice, wheat and RW production systems (Table S2). The EP indices can be used for assessing the crop production associated environmental effects^25^. About agro-ecosystem sustainability, earlier research findings have highlighted that EP indicator could be used to judge optimal land and crop management intensities^11,14^. This study suggests there is an enormous potential for enhancing the energy productivity and efficiency of RW system in the IGP. CSA scenarios (Sc4, Sc5 and Sc6) improved EUE and EP in rice, wheat as well as RW system, was due to lower energy input and higher energy output relative to Sc1. The findings of our research are in line with those who has described that CA-based management practices can reduce energy input and increase output^4,5,11,14^.

#### Yields, farm profitability and economic efficiency (Eco-efficiency)

**Figure 4:**
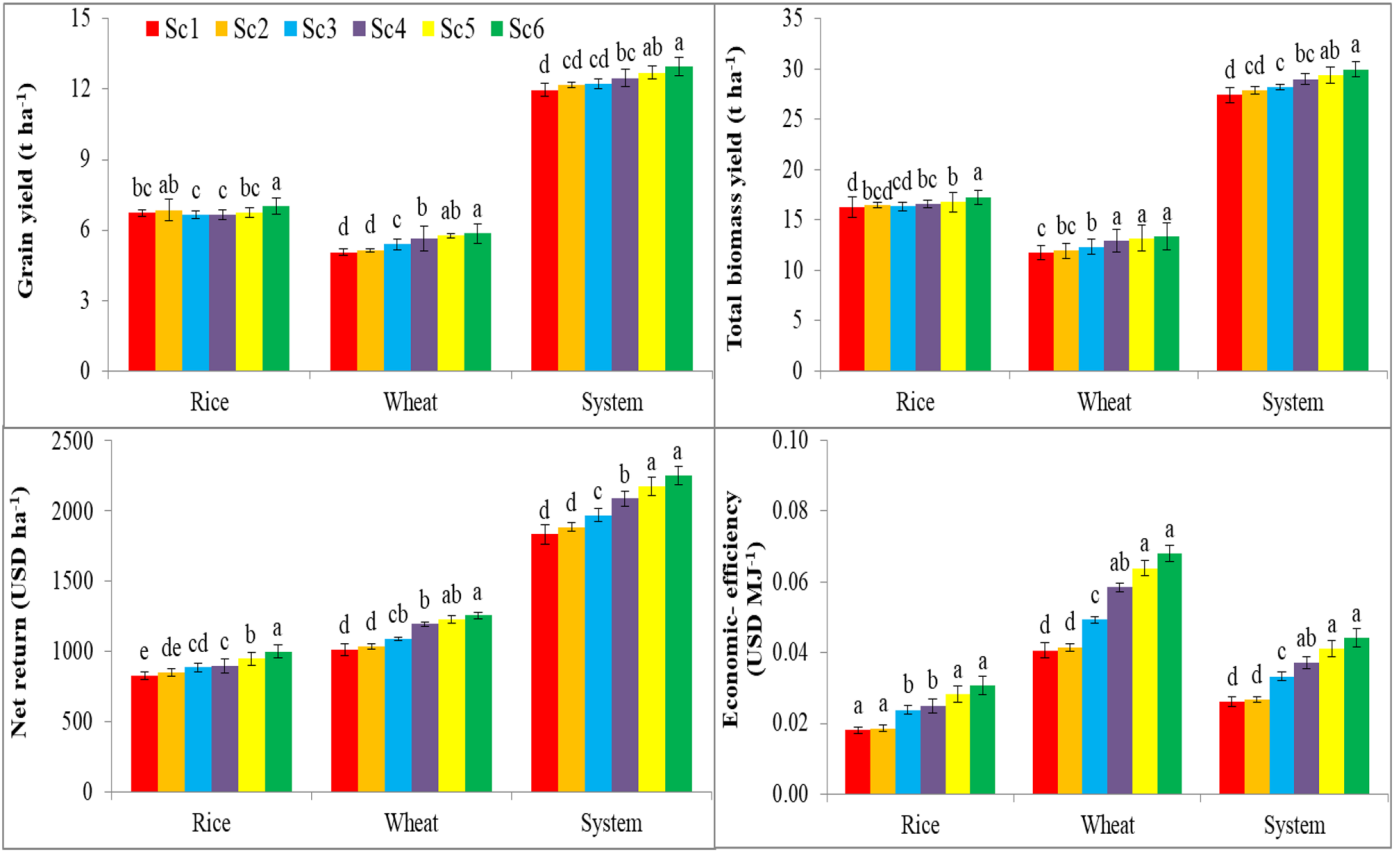
Effect of management practices portfolios on net return and eco-efficiency in rice, wheat and RW system (Mean of 3 years). Where; Sc1, business as usual-conventional tillage (CT) without residue; Sc2, CT with residue; Sc3, reduce tillage (RT) with residue + recommended dose of fertilizer (RDF); Sc4, RT/Zero tillage (ZT) with residue + RDF; Sc5, ZT with residue + RDF + GreenSeeker + Tensiometer; Sc6, Sc5 + Nutrient expert. Values with different lower case (a–e) letters are significantly different between each scenarios at *p* < 0.05 (Duncan multiple range tests for separation of mean). Vertical bars indicate ± S.E. of mean of the observed values.

The rice yields were not much influenced by different crop management. However, in wheat, CSAP (mean of Sc4, Sc5 and Sc6) produced 11–16% and 10–13% higher grain and biomass yield, respectively compared to BAU. The grain and biomass yield of RW system was improved by 4–8 and 6–9% under CSAP, respectively relative to Sc1 (3-years’ mean) (Fig. 4).The CSAP improved the net income of rice, wheat and RW system by 15, 21 and 23% (3-years’ mean), respectively relative to Sc1 (US$ 824 and 1009 and 1833 ha^−1^, respectively) (Fig. 4). Linear contrast effects were significant to the net income in rice, wheat and RW production systems (Table S2). Higher net income was associated with CSAP due to less cultivation cost in various crop production activities such as tillage, crop establishment and irrigation^9^. Researcher observed that escaping field operations particularly tillage puddling and manual transplanting in rice and adoption of ZTDSR minimized tillage and establishment costs by 79–85%. CSAP improved crop yields while reducing production costs resulting in greater profitability of the RW system.

The results showed that eco-efficiency varied from 0.018 to 0.031 US$ MJ^−1^ in rice and 0.041 to 0.068 US$ MJ^−1^ in wheat (Fig. 4). Overall, the eco-efficiency was the highest under CSAP in both the crops and the lowest under Sc1. Based on 3-years mean, CSAP recorded 56 and 57% higher eco-efficiency under rice and wheat than Sc1 (0.018 & 0.041 US$ MJ^−1^) (Fig. 4), respectively. Higher eco-efficiency of rice and wheat under CSAP (Sc4, Sc5 and Sc6) was due to lower energy input and more net returns in these scenarios as compared to Sc1. Linear contrast effects were significant to the eco-efficiency in rice, wheat and RW production systems (Table S2). Present study results suggested that eco-efficiency of RW system can be enhanced by the adoption of CSAP which can reduce the negative environmental impacts while at the same time can able to maintain or increase the farm returns^26^. Therefore, CSAP of RW systems with higher eco-efficiency are reflected as more economically and environmentally sustainable. Present finding also suggests that there is a huge potential exists for improving the eco-efficiency of RW production in the IGP.

#### Principal component analysis (PCA) and correlation

**Figure 5:**
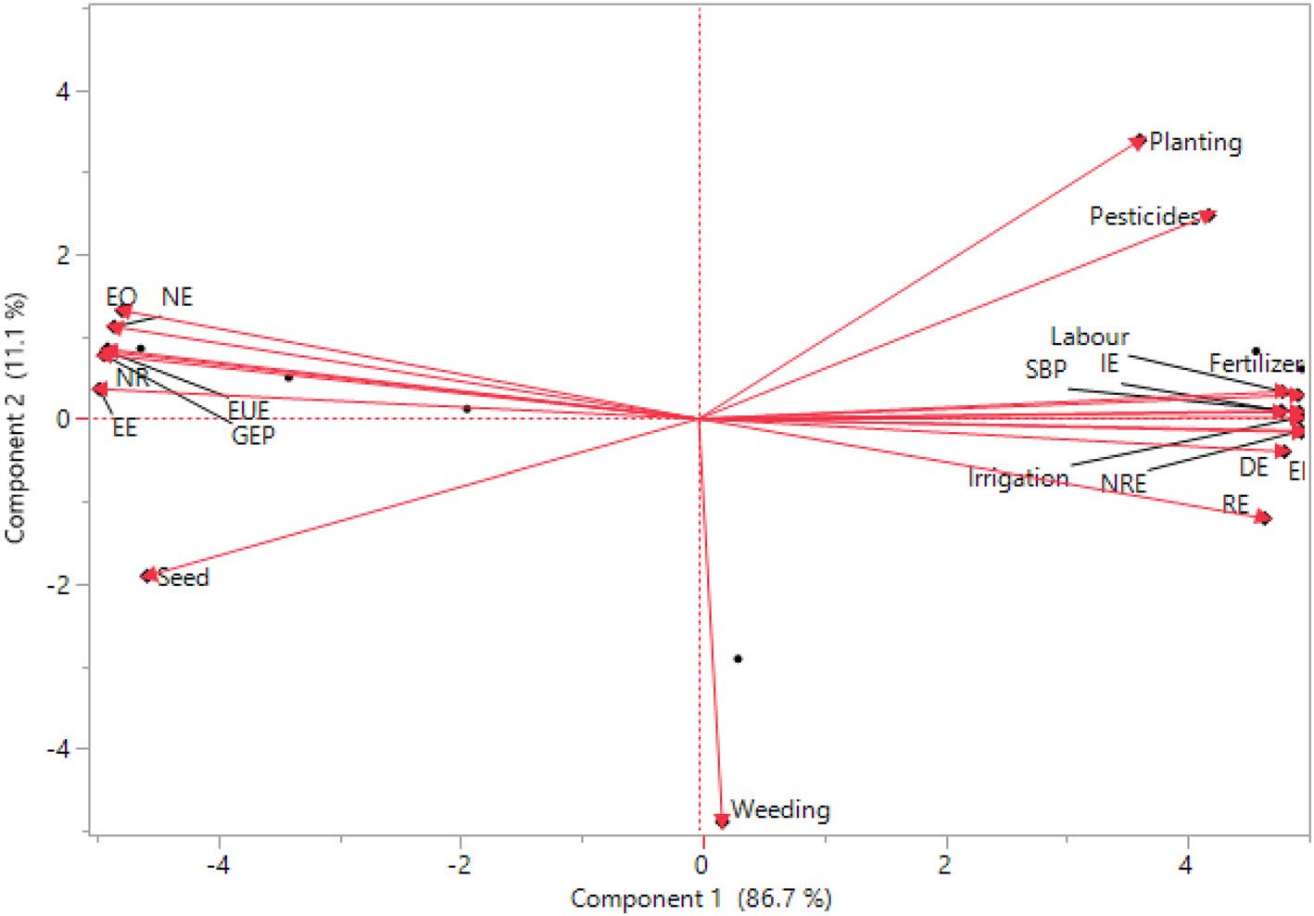
Principal component analysis among the energy and economic indicators under the RW system; Where; *SBP* seed bed preparation, *DE* direct energy, *IE* indirect energy, *RE* renewable energy, *NRE* non-renewable energy, *EI* energy input, *EO* energy output, *NE* net energy, *EUE* energy use efficiency, *GEP* grain energy productivity, *NR* net return, *EE* eco-efficiency.

**Figure 6:**
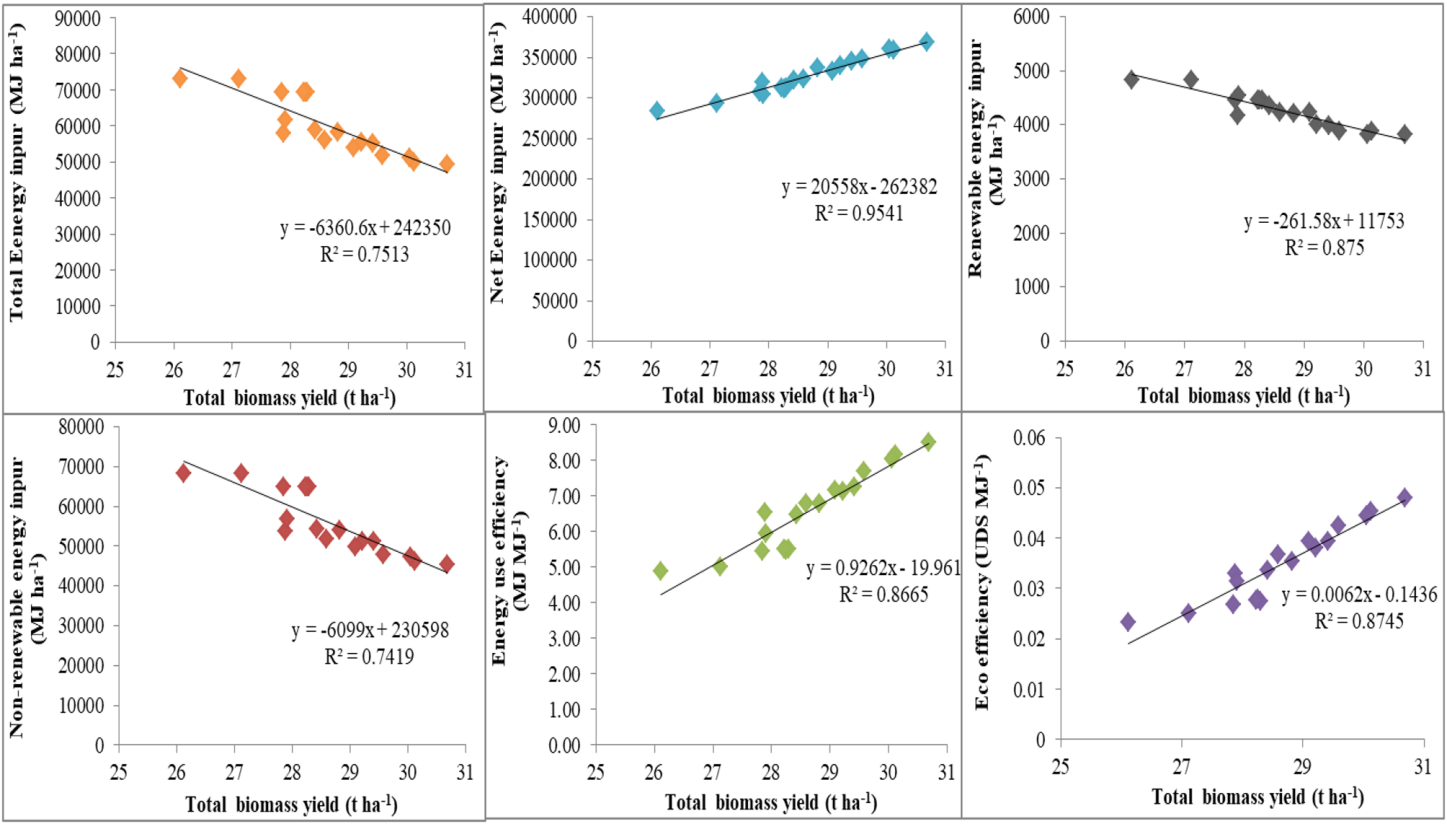
The relationship between the biomass yield and energy parameters (total energy inputs, net energy input, renewable and non-renewable energy inputs, energy and eco- efficiency).

Scatter plot of scenarios on PCA coordinates showed that BAU based scenarios are distinctly located on PCA coordinates (Fig. 5). Sc1, Sc2 and Sc3 scenarios are positioned in right-hand-side coordinates with higher weightage of PC1 (86.7% of total variance). A close association between energy parameters like fertilizer, irrigation, seed bed preparation, labour, sowing/planting, pesticides, direct energy, indirect energy, renewable energy, non-renewable energy, total energy inputs and net energy is also apparent from the PCA graph. The estimated energy components of various energy inputs were more under business as usual (BAU) due to higher energy use for tillage, irrigation, fertilizer use and pesticides in comparison to CSAP. However, the total energy inputs were lesser in CSAP (mean of Sc4, Sc5 and Sc6) followed by Sc3 and maximum in Sc1 and Sc2. This might be due to precise input management, proper crop establishment, efficient water management and efficient nutrient management. Layering of climate smart agriculture practices are significantly improves crop productivity and economics^2^. Present study results showed that the total energy inputs were correlated (positively or negatively) with energy output, net energy, EUE, EP, net return and eco-efficiency (Table S3). In particular, energy inputs were significantly negatively correlated with grain energy output (r = 0.96, *p* < 0.001) net energy (r = 0.97, *p* < 0.001), energy use efficiency (r = 0.98, *p* < 0.001), energy productivity (r = 0.98, *p* < 0.001), net return (r = 0.98, *p* < 0.001) and eco-efficiency(r = 0.99, *p* < 0.001) (Table S3). This strong correlation may be due to lower energy input and higher output energy^27^. Regression and Pearson’s correlation analysis were performed between the total biomass production and energy parameters (total energy inputs, net energy input, renewable and non-renewable energy inputs, energy and eco- efficiency) for validation purposes (Fig. 5). Total biomass yield of different management practices was significantly correlated to total energy inputs(R^2^ = 0.75, *p* < 0.001), net energy input (R^2^ = 0.95, *p* < 0.001), renewable energy (R^2^ = 0.88, *p* < 0.001), non-renewable energy (R^2 ^= 0.74, *p* < 0.001), energy use efficiency (R^2^ = 0.87, *p* < 0.001), and eco-efficiency (R^2^ = 0.87, *p* < 0.001) under different scenarios explaining their efficiency in predicting energy use efficiency (Fig. 6). Among different management practices, efficient use of energy inputs and higher energy use efficiency was associated with CSAP followed by improved management practices. Similarly^5^, researcher reported higher energy use efficiency in conservation agriculture based management practices over conventional rice–wheat system in north-west India.

## Conclusion

In our study, we attempted to explain the intricacies of energy input–output and energy flow of various management practices in the rice–wheat production system. In the study, it was found that rice–wheat production under business as usual (BAU) relies mostly on non-renewable energy sources; therefore, climate smart agriculture practices (CSAP) should be adopted to make use of available resources efficiently. Eco-efficiency of RW system was 57% higher under CSA scenarios compared to Sc1. A higher eco-efficiency reflects that CSAP are more economically and environmentally sustainable for RW system. Our results further showed that BAU practices under RW production in IGP of India is energy-input intensive as compared to CSAP. Thus, based on our study, we suggest that adopting CSAP portfolios can not only help in adapting to climatic risks but also provides viable tactic for enhancing energy output, energy use efficiency, eco-efficiency and keep productivity at its best. Energy management in RW production system should be considered as a key component in terms of efficient, sustainable and economical use of energy.

## Materials and methods

### Experimental site and climatic condition

A 3 years (2014–2017) on-farm study was carried out in three different climate smart villages i.e., Birnarayana (29° 75′ N, 76° 86 E), Anjanthali (29° 83′ N, 76° 88′ E) and Chandsamand (29° 80′ N, 77° 10′ E) at Karnal, India (Fig. S1). The climate of experimental sites are sub-tropical characterized by hot and dry summer and cold winters and receives nearby 70 cm annual rainfall, 80% of that occurs between June to September.

### Experimental details and management

The on-farm trials were initiated in the *rainy* season 2014, with six treatment combinations denoted as scenarios (Tables 4 and 5). The term scenario is a portfolio of agronomic practices where more than two agronomic interventions are used. Five different alternative scenarios related to, crop establishments, tillage, in-situ crop residue management, nutrient, and irrigation management were evaluated for business as usual practices (BAU) of the RW system. Six management scenarios (portfolios of management practices) were *i.e.,*Sc1-Business as usual(BAU)/Conventional tillage (CT) without residue, Sc2-CT with residue, Sc3-Reduce tillage (RT) with residue + recommended dose of fertilizer (RDF), Sc4-RT/Zero tillage (ZT) with residue + RDF, Sc5-ZT with residue + RDF + GreenSeeker + Tensiometer, Sc6-Sc5 + Nutrient expert were included (Tables 4 and 5). The varied portfolio of climate smart agriculture practices (CSAP) were layered in Sc4, Sc5 and Sc6. Sc1 and Sc2 are related to intensive tillage operation whereas Sc3 is related to reduce tillage operation with the traditional agronomic packages, and practices. Sc5 and Sc6 are related to no-tillage with modern agronomic packages and practices (Table 5). In scenario 6, the site-specific nutrient management (SSNM) approach was used to tailor the recommended nutrient doses using Nutrient Expert (NE) instead of RDF layered with Green Seeker guided N. Nutrient Expert is an interactive, computer-based decision-support tool that enables the implementation of SSNM in individual fields without soil test data^28^.

**Table 4:** Scenario notations and description of management protocols under different scenarios in rice–wheat (RW) system.

Scenarios		Tillage	Crop establishment	Laser land leveling	Residue management	Water management	Nutrient management	ICT
Name	Details							
Sc1	Business as usual (BAU)*or* Conventional tillage (CT) without residue	CT	TPR with random geometry. CTW using seed broadcasting	No	Residue removed	FP	FFP	None
Sc2	CT with residue	CT	TPR with random geometry. CTW using seed broadcasting	No	100% of rice and 25% of wheat residue incorporated	FP	FFP	None
Sc3	Reduce tillage (RT) with residue + recommended dose of fertilizer (RDF)	RT	DSR sown with MCP. RTW sown with RDD	No	Same as in Sc2	SR	RDF	None
Sc4	RT/Zero tillage (ZT) with residue + RDF	RT-ZT	DSR sown with MCP. ZTW sown with HS	Yes	100% rice residue retained and 25% wheat residue incorporated	SR	RDF	None
Sc5	ZT with residue + RDF + GreenSeeker + Tensiometer	ZT	DSR and ZTW sown with HS	Yes	100% of rice residue and 25% of wheat retained	Tensiometer based	RDF + GS guided N	Yes
Sc6	Sc5 + Nutrient expert	ZT	Same as in Sc5	Yes	Same as in Sc5	Tensiometer based	NE + GS guided N	Yes

*CT* conventional tillage, *RT* reduced tillage, *ZT* zero tillage, *TPR* transplanted rice, *CTW* conventional till wheat, *DSR* direct seeded rice, *MCP* multi crop planter, *RTW* reduced till wheat, *RDD* rotary disc drill, *ZTW* zero till wheat, *HS* happy seeder, *SR* state recommendation for irrigation, *FFP* farmer’s fertilizer practice, *RDF* recommended dose of fertilizer, *NCU* neem coated urea, *GS* green seeker, *NE* nutrient expert based fertilizer recommendation, *ICT* information and communication technology.

**Table 5:** Crop management practices for rice–wheat (RW) system under different scenarios.

Scenarios^a^/management practices	Sc1	Sc2	Sc3	Sc4	Sc5	Sc6
Field preparation	Rice—2 pass of harrow, 1 pass of rotavator, 2 pass of puddle harrow followed by (fb) planking; Wheat- 2 pass of harrow and rotavator each fb planking	Same as in Sc1	Rice-1 pass of harrow, 1 pass of cultivator fb planking; Wheat-1 pass of harrow, 1 pass of cultivator fb planking	Rice—Same as in Sc3; Wheat- Zero tillage	Zero tillage	Same as in Sc5
Seed rate (kg ha^−1^)^b^	Rice-12.5 kg and wheat100 kg	Same as in Sc1	Rice-20 kg and wheat-100 kg	Same as in Sc3	Same as in Sc3	Same as in Sc3
Crop geometry	Random geometry	Same as in Sc1	22–20 cm	Same as in Sc3	Same as in Sc3	Same as in Sc3
Source of fertilizers	Urea (46:0:0) and Di-ammonium phosphate (DAP) (18:46:0)	Same as in Sc1	Urea, DAP, Muriate of potash (MOP) (0:0:60), and NPK complex (12:32:16)	urea (46:0:0), DAP, MOP and NPK complex	Same as in Sc4	Neem coated urea (46:0:0), DAP, MOP and NPK complex
Fertilizer (N:P:K) in kg ha^−1^	Rice-195:58: 00; Wheat- 185:58:00	Same as in Sc1	Rice-150:60:60; Wheat- 150:60:60	Same as in Sc3	Rice-147:60:60 (in 1st year) 153:60:60 (in 2nd year) and 158:60:60 (in 3rd year); Wheat- 143:60:60 (in 1st year), 120:60:60 (in 2nd year) and 134:60:60 (in 3rd year)	Rice-138:39:70 (in 1st year), 140:42:57 (in 2nd year) and 145:44:57 (in 3rd year); Wheat-135:62:60 (in 1st year), 111:58:55 (in 2nd year) and 122:56:55 (in 3rd year)
Water management	Rice—Continuous flooding of 5–6 cm depth for 30–40 days after transplanting fb irrigation applied at alternate wetting and drying Wheat- 4–6 irrigation as per requirement	Same as in Sc1	Rice—Soil was kept wet up to 20 days after sowing fb irrigation applied at hair-line cracks Wheat- 4–6 irrigation as per critical crop growth stages	Same as in Sc3	Rice—Soil was kept wet till germination fb irrigation at − 20 to − 30 kPa matric potential; Wheat- Irrigation at − 50 to − 55 kPa matric potential	Same as in Sc5

^a^Refer Table 4 for scenario description.

^b^Seed treatment was done with Bavistin + Streptocycline @ 10 + 1 g per 10 kg seed-Raxil; Tebuconazole 2DS (2% w/w ) at 0.2 g a.i. kg^−1^ seed.

**Figure 7:**
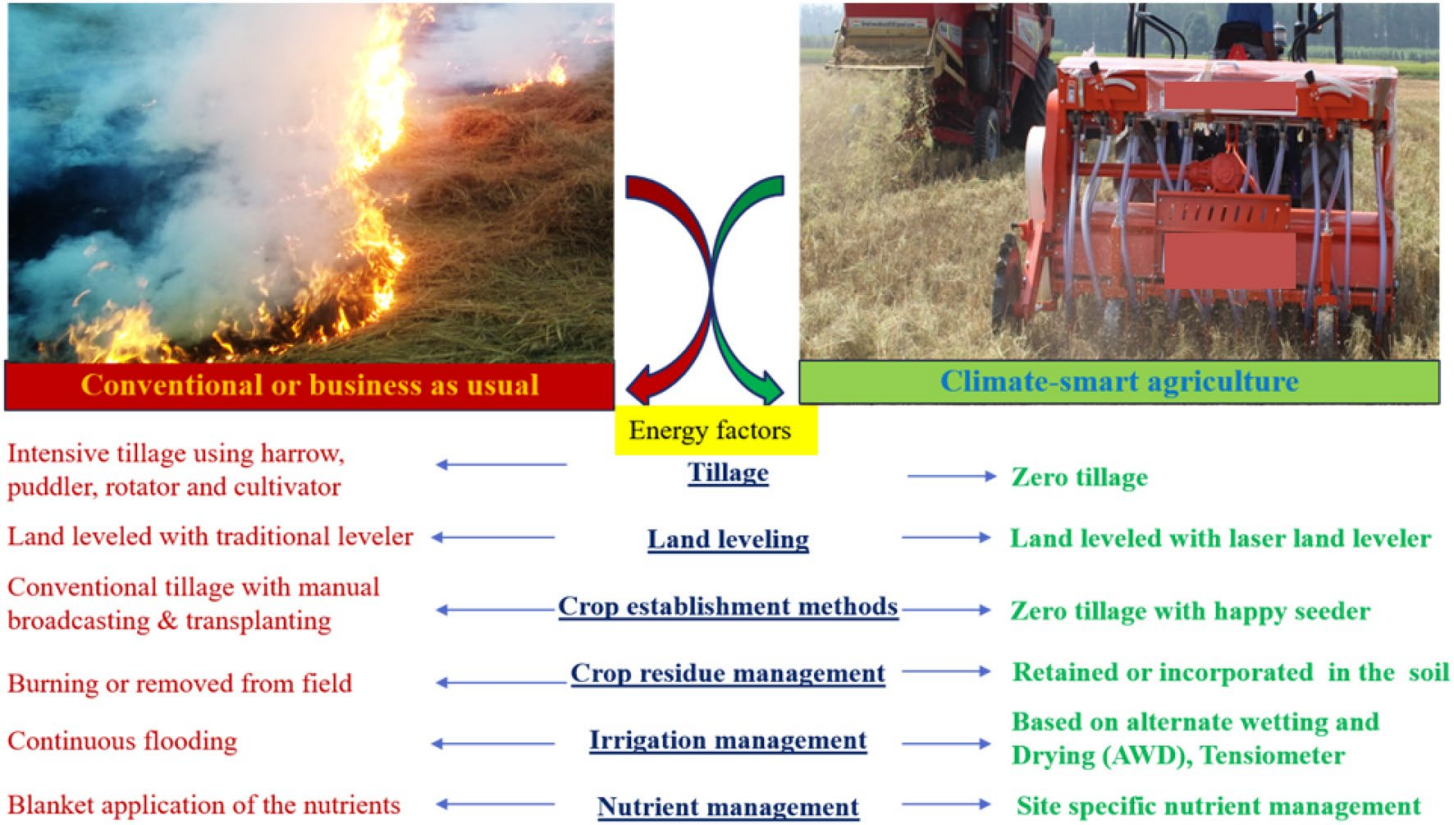
Energy sources of RW production under conventional management practice (left) and climate-smart agricultural practices (right).

All scenarios were evaluated in ~ 1000 m^19^ size of plot and it repeated at three locations. Energy`s sources of different management practices (conventional vs climate smart agriculture) are given in Fig. 7. Plant materials were handled according to relevant guidelines and regulations of CCS Haryana Agricultural University, Hisar, and ICAR-CSSRI, Karnal, India. The seeds of all the various crop varieties used in this study are readily available in India.

### Methods of energy analysis

#### Manual energy

For the determination of manual energy (ME) following equation was used:

1 $${\text{ME}}\left( {{\text{MJ}}} \right) \, = { 1}.{96 } \times {\text{ Lt }} \times {\text{ Ut}}$$

where Lt = Total no. of labour used in different farm operations.

Ut = Useful time consumed in different farm activities by a labour, h

Energy coefficients used for data analysis which are showed in Table 6. Manual labours were documented in each farm activities with working hours that was transformed in man-hours.

**Table 6:** Energy equivalents used in the study for different agricultural operations.

Particulars	Units	Energy coefficients (MJ Unit^−1^)	References
**Input**			
Human labour	Man-hour	1.96	^22^
Diesel	Litre	56.31	^22^
Nitrogen (N)	Kg	66.14	^22^
Phosphorus (P_2_O_5_)	Kg	22.44	^22^
Potassium (K_2_O)	Kg	11.15	^22^
Herbicides, insecticides and pesticides	Kg	120.00	^22^
Irrigation water	ha-cm	143.56	^22^
Zinc sulphate (ZnSO_4_)	Kg	8.40	^12^
Iron sulphate (FeSO_4_)	Kg	110.00	^12^
Rice and wheat seed	Kg	14.70	^22,12^
Tractor	Kg	93.61	^22^
Other machinery	Kg	62.70	^22^
Combine harvester	Kg	87.63	^22^
**Output**			
Rice and wheat seed	Kg	14.70	^22,12^
Rice and wheat straw	Kg	12.50	^22,12^

#### Mechanical/power-driven energy

Indirect energy of agricultural machineries was estimated on the basis of total diesel consumed during the seedbed preparation, sowing of crops, harvesting, threshing and transportation, etc. (Table S1)^9,14,26^. The total time consumed in different operations was also recorded during different farm activities. Total fuel energy was estimated based on the consumption of diesel in various farm operations by using the following equation:

2 $${\text{ME}} = \frac{{{\upbeta } \times {\upmu }}}{{{\upgamma } \times {\upalpha }}}$$

here ME—machinery energy (MJ ha^−1^), β—energy conversion factor for machinery (MJ kg^−1^), µ—machinery weight (kg), γ—effective field capacity (ha h^−1^) and α—life of the machinery (h).

Effective field capacity (γ) was computed by using the following equation:

3 $${\text{EFC}} = \frac{{\text{A}}}{{\text{T}}}$$

where EFC—effective field capacity, A—indicates total area covered (ha) and T—time require (h).

The fuel energy was computed with the following equation:

4 $${\text{FE }} = { 56}.{\text{31 D MJ}}$$

where FE—Fuel energy (MJ ha^−1^), 56.31 is energy coefficient of diesel (MJ L^−1^) D—total diesel consumed, L

#### Irrigation energy

The energy required to pump out the water from the bore wellswas calculated on the basis of following equation:

5 $${\text{DE}} = \frac{{{\upalpha } \times {\upbeta } \times {\upgamma } \times {\upmu }}}{{{\text{y }} \times {\text{ z}}}}$$

where DE—Direct energy (MJ ha^−1^), α—Density of water (1000 kg m^−3^), β—Acceleration due to gravity (9.80 m s^−2^), γ-Depth of dynamic head (m), µ–Seasonally volume of water needed (m^3^ ha^−1^), y–Pump’s efficiency (80%) and z–Efficiency of power conservation (20%)^20^. For estimation of irrigation energy, transmission and production efficiencies were also involved.

#### Inputs energy

Input energy (expressed in MJ ha^−1^) of each interventions were calculated based on study of other researchers^4,9,14^. Basic information on energy inputs (like, tillage, fertilizers, pesticides, irrigations, harvesting and threshing, transportation and other crop management activities) and outputs (in terms of rice and wheat yields) were entered into excel spreadsheets. Energy inputs under different management scenarios were calculated by multiplying the inputs with the equivalent energy coefficients (Table 6). The term Indirect-energy indicates to energy used in various forms/activities such as manufacturing, packaging, and transporting of machinery, chemical fertilizers and pesticides, whereas the term direct-energy refers to the energy consumed in the various forms such as diesel, human labour, tractor and electricity. The direct and indirect energy coefficients are presented in Table 6 which took derived from peer-reviewed literature. Below given equations are used for calculation of the following energy parameters^4,19,22,29^(Table 7).

**Table 7:** Equations for calculation of energy parameters.

Energy types	Components/factors	Unit
Direct energy	Diesel + labour + tractor + electricity	(MJ ha^−1^)
Indirect energy	Machinery + fertilizers + pesticides + seeds	(MJ ha^−1^)
Renewable energy	Labour + tractor + seeds	(MJ ha^−1^)
Non-renewable energy	Machinery + diesel + electricity + chemical fertilizer + pesticides	(MJ ha^−1^)
Total energy input	Direct/renewable + indirect/non-renewable energy	(MJ ha^−1^)
Grain energy output	Energy in the harvested grain (grain)	(MJ ha^−1^)
Total energy output	Energy in the harvested total biomass (grain + straw)	(MJ ha^−1^)
Net energy	Total energy output—energy input	(MJ ha^−1^)
Energy use efficiency	Total energy output/energy input	(MJ MJ^−1^)
Energy productivity	Grain yield/energy input	(kg MJ^−1^)

#### Economic-efficiency (Eco-efficiency)

The term eco-efficiency is an index facilitating adequate de-linking of the use of available natural resources from economic activity or pollutant release from economic activity required to meet human needs. It can be defined as a ratio between economic value-added and an environmental degradation^26,30,31^. Eco-efficiency of the RW system can be improved by choice climate smart agriculture (CSA) which decreases negative environmental impacts while at the same time maintaining or increasing farm returns^32^. Therefore, agricultural production systems with higher eco-efficiency are considered more economically and environmentally sustainable. The main target of CSA is to improve its EE by reducing agriculture’s environmental footprints (eg. energy use & emission of GHGs) while increasing farm profit^17,26^.

Eco-efficiency refers to the efficiency of economic motion in relation to its effect on the environment. The environmental effect can be measured in terms of the total quantity of greenhouse gas emitted (kg CO_2_ eq.) or energy used (MJ) or by various practices of farming. In this paper, eco-efficiency was computed by using the following equation:

6 $${\text{Eco}} - {\text{efficiency }}\left( {{\text{USD MJ}}^{ - 1} } \right) = \frac{{{\text{Economic Return }}\left( {{\text{USD ha}}^{ - 1} } \right)}}{{{\text{Environmental Impact }}\left( {{\text{MJ ha}}^{ - 1} } \right)}}$$

#### Statistical analysis

This on-farm adaptive research experiment was conducted for consecutive 3-years in a randomized block design (RBD) with three replications. All agronomic data were recorded during field experimentation and were analysed using the analysis of variance (ANOVA) technique^33^. Data analysis was done with the help of SAS 9.1 software^34^. Tukey’s honestly significant difference (HSD) method was used for comparing treatment means at 5% level of significance. Principal component analysis (PCA) was done with JMP 14.1 software. The results were submitted to PCA in order to determine the common relationships between parameters.

## Supplementary Information

Supplementary Information.
