# Supplementary material for: Energy and economic efficiency of climate-smart agriculture practices in a rice–wheat cropping system of India
Source: Sci Rep. 2022 May 24;12:8731. doi: 10.1038/s41598-022-12686-4 (PMC9130264; doi:10.1038/s41598-022-12686-4)
Supplement: Supplementary file 1 — Supplementary Information. [file 41598_2022_12686_MOESM1_ESM.docx]

**Supplementary files**

Table **S1**

Operational parameters of implements used in different mechanical operations of RW system

| `Implements | Harrow | Planker | Cultivator | ZT seed drill | Turbo Happy Seeder | Rotavator | Rotary drill |
| --- | --- | --- | --- | --- | --- | --- | --- |
| Operating speed (km h^-1^) | 9.60 | 13.60 | 11.90 | 9.10 | 6.60 | 5.70 | 5.90 |
| Working depth (cm) | 10.50 | -NA- | 15.50 | 5.85 | 4.50 | 12.00 | 5.02 |
| Theoretical width (m) | 1.73 | 4.81 | 2.76 | 1.64 | 1.19 | 1.24 | 1.73 |
| Working width (m) | 1.80 | 3.54 | 2.32 | 1.80 | 1.80 | 2.18 | 2.50 |
| Effective ﬁeld capacity (h ha^-1^) | 0.92 | 1.85 | 1.41 | 0.83 | 0.44 | 0.33 | 0.34 |
| Fuel consumption (l h^-1^) | 10.01 | 4.40 | 8.80 | 7.50 | 15.80 | 14.90 | 10.50 |
| Field efﬁciency (%) | 53.03 | 38.30 | 51.00 | 51.07 | 37.21 | 26.33 | 23.15 |

**Table S2.** Significance effect of management practices portfolios and their linear contrast on energy indicators, net return, and eco-efficiency under rice, wheat and system.

| Scenarios^a^ | Direct energy | Indirect energy | Renewable energy | Non-Renewable energy | Total energy input | Total energy output | Net energy | Energy use efficiency | Grain energy productivity | Net Return | Eco-effciency |
| --- | --- | --- | --- | --- | --- | --- | --- | --- | --- | --- | --- |
| ***Rice*** | | | | | | | | |  |  |  |
| BAU *vs* CSAP | ** | *** | * | *** | *** | NS | NS | * | ** | * | ** |
| BAU vs I-BAU | NS | *** | NS | * | NS | NS | NS | NS | NS | NS | NS |
| I-BAU *vs* CSAP | ** | *** | NS | ** | ** | NS | NS | * | * | NS | * |
| ***Wheat*** | | | | | | | | |  |  |  |
| BAU *vs* CSAP | *** | *** | * | *** | *** | *** | *** | *** | ** | ** | *** |
| BAU *vs* I-BAU | NS | ** | NS | ** | ** | NS | * | * | NS | NS | NS |
| I-BAU *vs* CSAP | *** | *** | ** | *** | *** | *** | *** | *** | *** | ** | *** |
| ***System*** | | | | | | | | |  |  |  |
| BAU vs CSAP | *** | *** | * | *** | *** | NS | * | ** | ** | ** | ** |
| BAU *vs* I-BAU | NS | ** | * | *** | ** | NS | NS | NS | NS | NS | NS |
| I-BAU *vs* CSAP | *** | *** | NS | ** | *** | NS | * | ** | * | ** | ** |

**Where:** BAU-Business as usual (Sc1), I-BAU; Improved-Business as usual (Sc2 and Sc3) and CSA-Climate smart agricultural practices (Sc4, Sc5 and Sc6);

**Table S3.** Significant (p < 0.05) linear correlation indices between variables used in the principal component analysis

| Energy and economic indicators | Direct energy | Indirect energy | Renewable energy | Non-renewable energy | Total energy input | Total energy output | Net energy | Energy use efficiency | Grain energy productivity | Net Return | Eco-efficiency |
| --- | --- | --- | --- | --- | --- | --- | --- | --- | --- | --- | --- |
| Direct energy | 1.0000 |  |  |  |  |  |  |  |  |  |  |
| Indirect energy | 0.8785 |  |  |  |  |  |  |  |  |  |  |
| Renewable energy | 0.99*** | 0.84* |  |  |  |  |  |  |  |  |  |
| Non-Renewable energy | 0.97** | 0.96** | 0.94** |  |  |  |  |  |  |  |  |
| Total energy input | 0.97** | 0.97** | 0.94** | 1.0000 |  |  |  |  |  |  |  |
| Total energy output | -0.93** | -0.93** | -0.94** | -0.96** | -0.96** |  |  |  |  |  |  |
| Net energy | -0.94** | -0.94** | -0.95** | -0.97** | -0.97*** | 0.99*** |  |  |  |  |  |
| Energy use efficiency | -0.93** | -0.97** | -0.92** | -0.98*** | -0.98*** | 0.99*** | 0.99*** |  |  |  |  |
| Grain energy productivity | -0.96** | -0.96** | -0.95** | -0.98*** | -0.98*** | 0.99*** | 0.99*** | 0.99*** |  |  |  |
| Net Return | -0.94** | -0.96** | -0.94** | -0.98*** | -0.98*** | 0.99*** | 0.99*** | 0.99*** | 0.99*** |  |  |
| Eco-effciency | -0.95** | -0.98*** | -0.93** | -0.99*** | -0.99*** | 0.97** | 0.98*** | 0.99*** | 0.99*** | 0.99*** | 1.0000 |

**
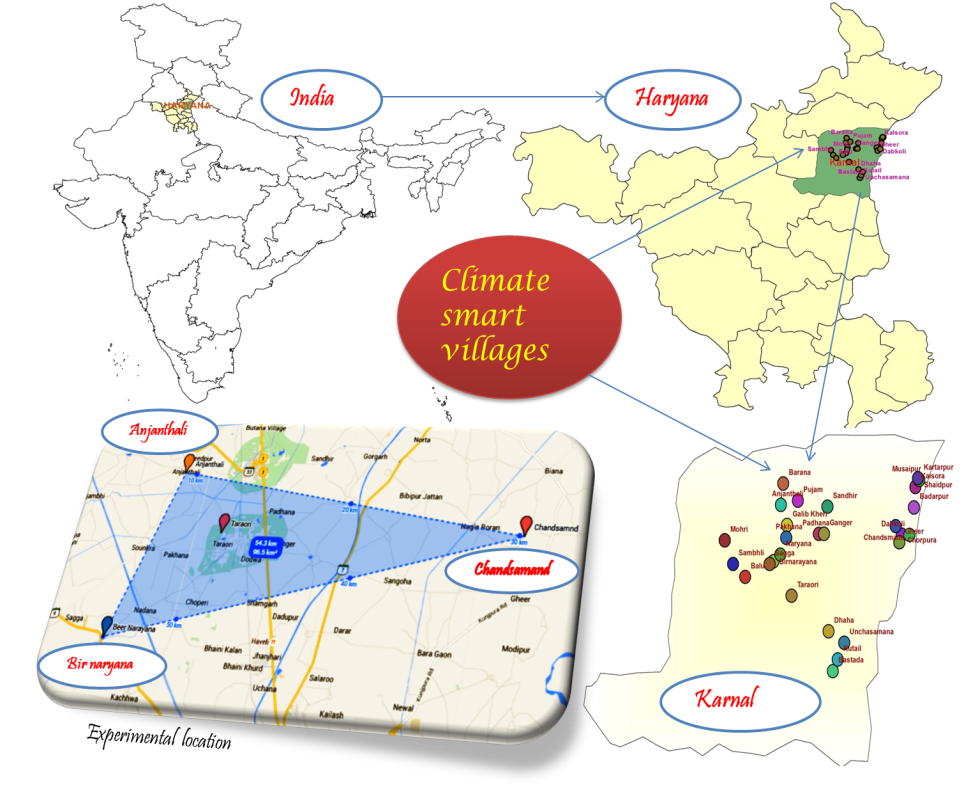
**

Fig.S1. Location of experimental sites in Karnal districts of Haryana, India. (Figure created by the author; SK Kakraliya; The ArcGis software has used to create the map in the figure (2019), version (10.71)^37^.


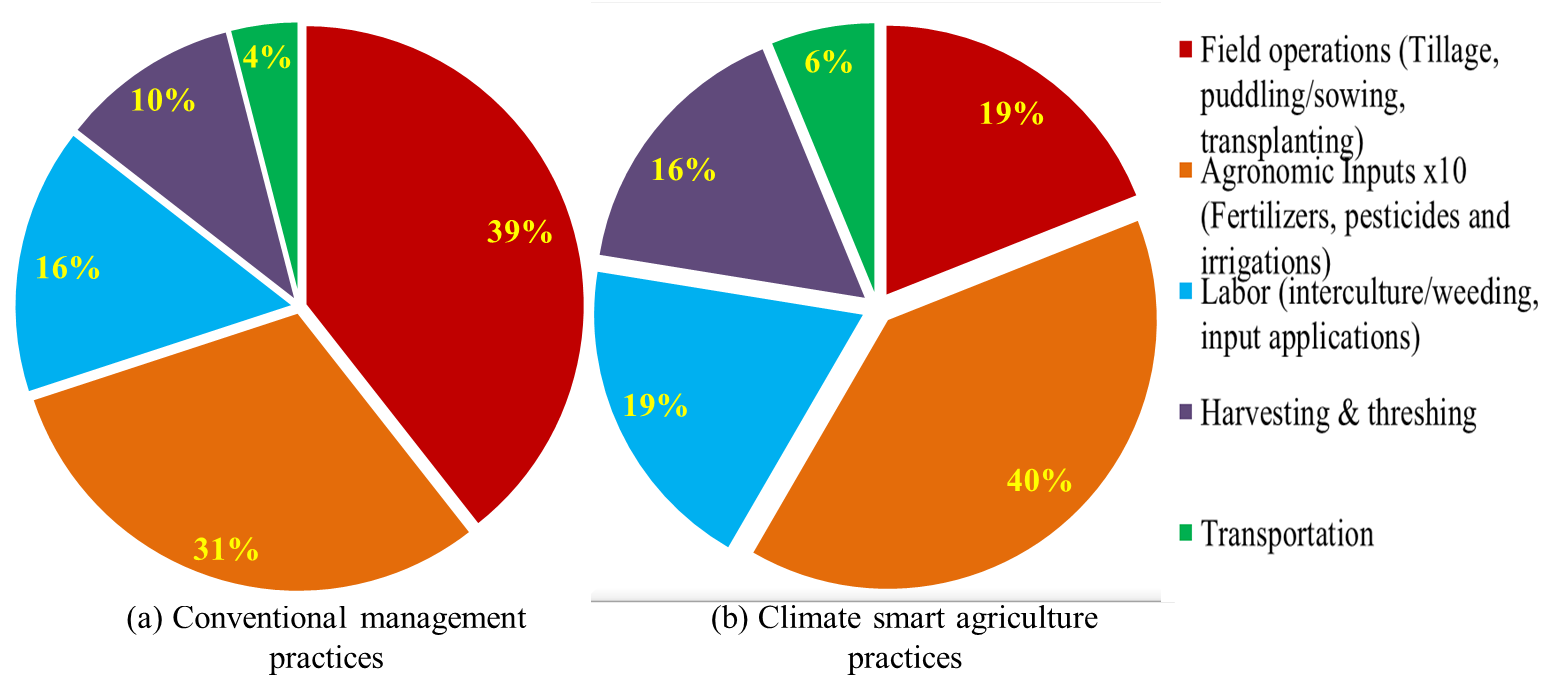


Fig. S2. Operation- and source- wise total energy utilization pattern (%) of R-W system under BAU (Sc1) (a) *versus* CSA practices (mean of Sc4, Sc5 and Sc6) (b)
